# Feature fusion method for pulmonary tuberculosis patient detection based on cough sound

**DOI:** 10.1371/journal.pone.0302651

**Published:** 2024-05-14

**Authors:** Wenlong Xu, Xiaofan Bao, Xiaomin Lou, Xiaofang Liu, Yuanyuan Chen, Xiaoqiang Zhao, Chenlu Zhang, Chen Pan, Wenlong Liu, Feng Liu

**Affiliations:** 1 College of Information Engineering, China Jiliang University, Hangzhou, Zhejiang, China; 2 Hangzhou Red Cross Hospital, Hangzhou, Zhejiang, China; 3 School of Information Technology and Electrical Engineering, University of Queensland, Brisbane, Queensland, Australia; University of Aizu, JAPAN

## Abstract

Since the COVID-19, cough sounds have been widely used for screening purposes. Intelligent analysis techniques have proven to be effective in detecting respiratory diseases. In 2021, there were up to 10 million TB-infected patients worldwide, with an annual growth rate of 4.5%. Most of the patients were from economically underdeveloped regions and countries. The PPD test, a common screening method in the community, has a sensitivity of as low as 77%. Although IGRA and Xpert MTB/RIF offer high specificity and sensitivity, their cost makes them less accessible. In this study, we proposed a feature fusion model-based cough sound classification method for primary TB screening in communities. Data were collected from hospitals using smart phones, including 230 cough sounds from 70 patients with TB and 226 cough sounds from 74 healthy subjects. We employed Bi-LSTM and Bi-GRU recurrent neural networks to analyze five traditional feature sets including the Mel frequency cepstrum coefficient (MFCC), zero-crossing rate (ZCR), short-time energy, root mean square, and chroma_cens. The incorporation of features extracted from the speech spectrogram by 2D convolution training into the Bi-LSTM model enhanced the classification results. With traditional futures, the best TB patient detection result was achieved with the Bi-LSTM model, with 93.99% accuracy, 93.93% specificity, and 92.39% sensitivity. When combined with a speech spectrogram, the classification results showed 96.33% accuracy, 94.99% specificity, and 98.13% sensitivity. Our findings underscore that traditional features and deep features have good complementarity when fused using Bi LSTM modelling, which outperforms existing PPD detection methods in terms of both efficiency and accuracy.

## Introduction

The World Health Organization reports that Tuberculosis (TB) ranks as the second most fetal infectious disease following the corona virus disease of 2019 (COVID-19) and is the 13th leading cause of death in the worldwide. TB is also the "number one killer" of people living with HIV [1]. Most TB cases are recorded in developing and undeveloped countries, where adequate medical facilities for tuberculosis screening and treatment are often unaffordable [2]. Delayed diagnosis, or failure to receive diagnosis and treatment exacerbates the spread of tuberculosis, imposing a heavier social burden. In South Africa, poor citizens experience an average of 33-days gap between the first TB symptoms and the first TB test, for poorer citizens, this interval increases to 90 days, and one untreated infectious TB patient can infect 10 - 15 people per year [3]. Early diagnosis is vital for the treatment of TB and the control of transmission. Xpert MTB/RIF, Interferon Gamma Release Assay (IGRA), and Purified Protein derivative (PPD) are currently the most used screening methods for TB. Xpert MTB/RIF and IGRA have high accuracy, however they are complex in operation, expensive, and require special laboratory facilities [4–7]. Thus the methods are unsuitable for large-scale screening. PPD is usually used for community screening due to its simplicity and low cost. Unfortunately, the average sensitivity and specificity of PPD are only 77% and 79% respectively. A more critical flaw is that the test results are susceptible to the BCG vaccine, which can lead to higher total costs of screening [8–10]. An accurate, simple, and economical screening method is crucial for the timely diagnosis and control of pulmonary tuberculosis.

Research on the diagnosis of respiratory diseases based on audio signals, such as breath sounds, voice, and cough sounds, has always been received significant attention, especially since the outbreak of COVID-19. Owing to its non-contact collection characteristics, research focusing on the analysis of cough sounds has experienced the most rapid growth. Auscultation-based respiratory sounds are traditional clinical diagnostic methods. Sound can reflect changes in lung tissues, organs, bronchus secretions, and carry signals related to lung and respiratory abnormalities. Deep learning has been widely used in medical-related classification tasks, particularly in the classification of tumors [11,12]. The diagnosis of various types and severities of respiratory diseases can be facilitated by analyzing autonomous or stimulated human sounds, such as breathing and coughing [13]. Breath sounds and voice analysis have been used in diagnosing Chronic Obstructive Pulmonary Disease (COPD) [14], corona virus disease (COVID-19) [15], and chronic lung virus infections [16]. In addition, cough sounds have been used to discriminate COPD and asthma [17–19] in children [20,21], and diagnose TB [2], and COVID-19 [22–24].

### The present research

The present study analyzed cough data from hospitals to: i) verify whether pulmonary tuberculosis screening can be achieved based on traditional signal features of cough sounds, ii) explore which model can achieve better detection and classification performance, iii) verify whether the traditional characteristic signals of cough sounds are complementary to the speech spectrogram, and IV) explore whether AI models based on feature fusion are expected to achieve more effective pulmonary tuberculosis screening than current PPD methods.

## Related work

### Screening for lung disease based on acoustic

Recently acoustic signals have been widely used for the screening and evaluation of lung diseases. Compared with traditional radiography, CT, spirometry, and bronchoscopy, it is simpler and less expensive to perform and can reduce exposure, thereby helping to prevent the spread of infectious diseases. Grant [15] proposed a method to detect coronary pneumonia using speech and breath sounds by extracting the Mel Frequency Cepstrum Coefficient (MFCC) and RASTA-PLP features, with 81 patients with coronary pneumonia and 1118 patients without COVID-19, using random forest and deep neural networks. This resulted in the optimal detection of speech and mutual trust, with an AUC of 0.7938 for speech detection and 0.7575 for breath sound detection. Khan [16] used breath sounds to classify chronic lung viruses by extracting five non-linear dynamic system features and inputting them into a K-nearest neighbor classifier. They obtained 99.4% accuracy, 99.99% sensitivity, 97.82% specificity, and 99% precision after 5-fold cross-validation.

### Screening for lung disease based on cough sound

Cough is the most common symptom of many respiratory diseases. Combined with artificial intelligence technology, especially deep learning, there is increasing research on classifying lung diseases based on cough sounds. Features of cough sound signals such as non-Gaussianity, zero-crossing rate (ZCR), Mel Cepstra, MFCC, zero-crossing irregularity, and symptoms are widely used for the screening of lung diseases. In addition, different artificial intelligence models, such as logistic regression classifiers (LR) and support vector machines (SVM), are selected according to the number of samples and features. Long short-term memory networks (LSTM) and bidirectional long short-term memory networks (Bi-LSTM) are typically used to classify audio signals. GoogleNet, ResNet18, ResNet50, ResNet101are models which are commonly used in image and spectrogram-based classification. Infante [17] extracted the zero crossing irregularity and rate of decay features of cough sounds and then used an LR model to classify patients with different respiratory diseases. They obtained an AUC of 0.94. Liao [13] extracted MFCC features and used an SVM to achieve an accuracy of up to 86.04% and standard deviation of 4.7% for lung disease classification. Balamurali [20] extracted MFCCS and used Bi-LSTM to identify asthma, upper respiratory tract infections, and lower respiratory tract infections. Loey [23] transformed speech into a speech spectrogram and used GoogleNet, ResNet18, ResNet50, ResNet101, MobileNetv2, and NasNetmobile models to screen for new coronary pneumonia, and finally obtained 94.44% sensitivity and 95.37% specificity with ResNet18. Abeyratne [25] extracted non-Gaussianity and Mel Cepstra and obtained a sensitivity of 94% and specificity of 75% using LR.

### Tuberculosis screening based on cough sounds

Tuberculosis is an infectious disease. Botha [3] classified 17 patients with TB and 21 healthy subjects by extracting MFCC features in cough sounds with an accuracy of 78% and an AUC of 0.94. LR. Pahar [26] classified 16 TB patients and 35 healthy subjects with 95% specificity and 93% sensitivity. Pahar [27] differentiated 47 patients with TB, 229 patients with neo conjunctivitis, and 1498 healthy subjects using the CNN, LSTM, and Resnet50 models. The best result was achieved in the Resnet50 model, with 92.59% accuracy with a model in differentiating TB from COVID-19, and 86.31% in accuracy with triple classification. Frost [2] used a modified Bi-LSTM to classify cough sounds in 28 patients with TB and 46 healthy subjects using mel-spectrograms, linear filter-bank energies, and MFCCs. Although all these studies have stimulated and encouraged the possibility of using cough sounds for the diagnosis of pulmonary tuberculosis, to the best of our knowledge, the literature so far often has two shortcomings: (1) the amount of tuberculosis patient data used was less than 50 cases and (2) only traditional feature-based analysis or machine learning methods were mainly used.

## Methods

**Fig 1:**
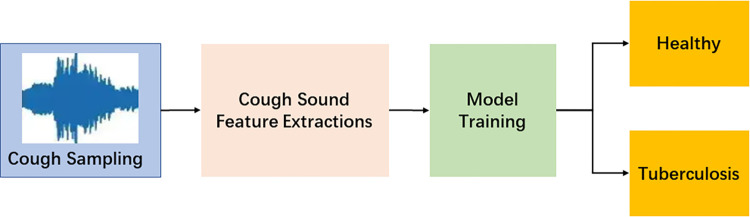
General feature classification.

**Fig 2:**
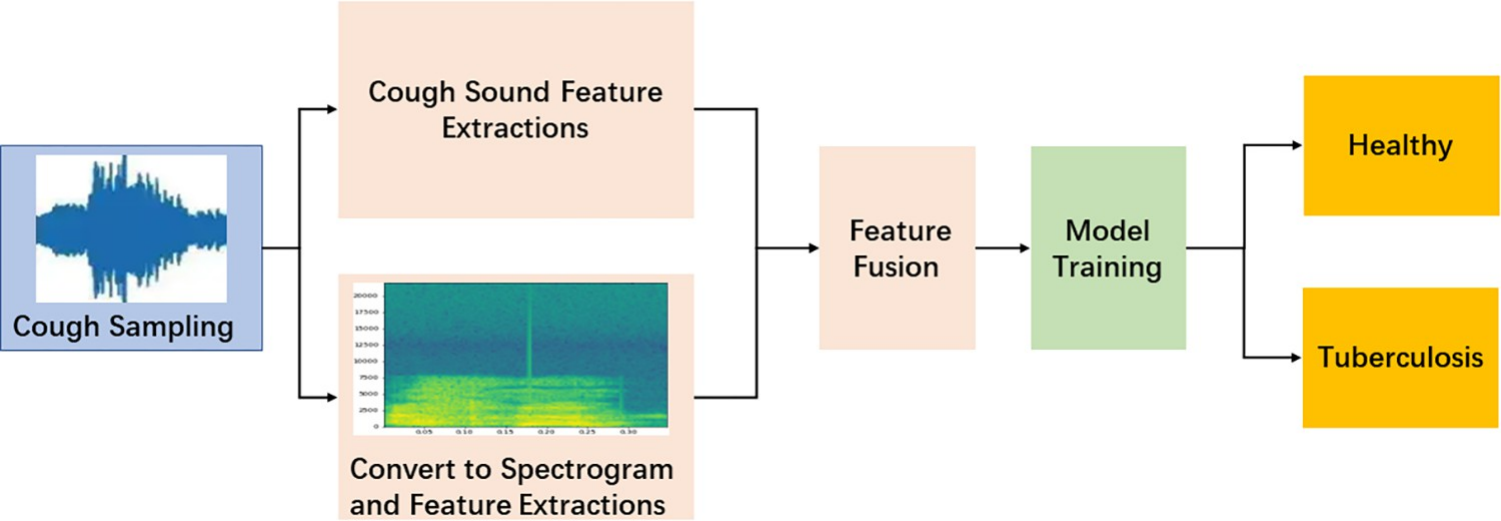
Feature fusion classification process.

The experimental information before and after feature fusion is shown in **Figs 1** and **2**. The experiment in **Fig 1** uses traditional feature extraction methods for classification. The experiment in **Fig 2** combines the features obtained through training from spectrograms and the deep features obtained through training from traditional features, This combined approach utilizes a classification layer for classification purposes.

### Dataset

**Fig 3:**
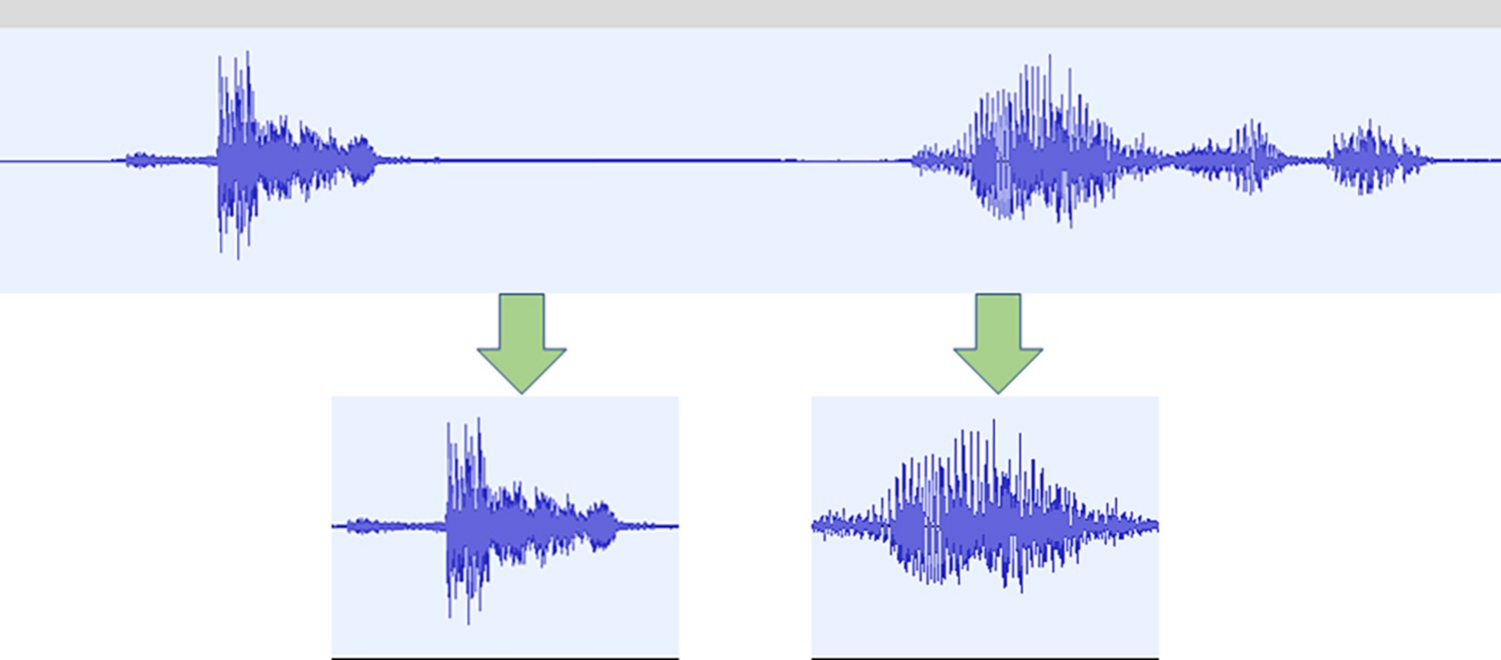
Cough tone-cutting process.

The dataset was collected using a smartphone at the Hangzhou Red Cross Hospital, Hangzhou, Zhejiang, China. The study was approved by the Ethics Committee of Hangzhou Red Cross Hospital (approval no. [2023] (025) at 2023.2.20). The cough sounds, sex, age, and weight of each subject were collected. Data Desensitization Processing was performed on the collected data and differentiated patients with specialized numbers. The microphone of the mobile device was placed approximately 30 to 40 cm away from the mouth during the collection, and the mobile device was angled upwards at approximately 45°. The data collection process was conducted in a quiet environment under the guidance of a doctor onsite. Each participant was required to cough three or more times, with sufficient time intervals between every two to ensure that each cough was fully aspirated. A total of 144 cough sound files were collected from 70 patients with confirmed TB and 74 healthy individuals. The composition of the data objects is presented in **Table 1**. The average duration of each cough sound recording was 20 seconds. The Audacity software was used to extract 456 cough sound segments from 144 cough sound records, with each cough sound segment lasting 0.35 seconds. The process is shown in **Fig 3**, and the composition of the cough sound segments is listed in **Table 2**. The ratio of the training set to the test set was 8:2.

**Table 1:** Gender and age composition of the dataset.

	Number of Subjects	Gender (Male: Female)	18-40 (Age)	41-65 (Age)	>=66 (Age)
**Tuberculosis**	70	45:25	16	30	24
**Healthy**	74	37:37	17	33	24

**Table 2:** Clipping of cough sound clip.

	Number of Cough Records	Number of Cough Sound Segments
**Tuberculosis Positive**	**70**	**230**
**Healthy**	**74**	**226**
**Total**	**144**	**456**

### Feature extraction

In the literature, MFCC and ZCR have been widely used for cough sound classification. The experiment divided each cough sound segment into 31 frames, extracted 44 MFCCs, and one over-zero rate per frame. Then, the Bi-LSTM model was applied for training and classification. The results show that it cannot achieve good classification results with MFCC and ZCR features. Subsequently, the features of the short-time energy (TSE), RMS, and chroma_cens were added individually in the experiment **Table 3**. The speech spectrogram of each cough sound fragment was generated and imported for classification using a convolutional neural network [28]. Finally, we proposed a method to fuse the features extracted by both approaches [29], and then trained them with recurrent neural networks and convolutional neural networks, respectively, and fused the deep features obtained after training.

**Table 3:** Result of the feature selection experiment.

Features	Accuracy	Specificity	Sensitivity
M,Z	88.04%	86.96%	88.89%
M,Z,T	89.13%	93.33%	84.78%
M,Z,R	89.15%	84.80%	93.33%
M,Z,C	90.50%	91.39%	90.65%
M,Z,T,R	90.22%	91.11%	89.13%
M,Z,T,C	92.30%	93.18%	91.49%
M,Z,C,T,R	**93.99%**	**93.93%**	**92.39%**

where M is MFCC, Z is ZCR, T is the short time energy, R is the RMS, C is the Chroma_cens.

#### MFCC extraction

With the steps of preemphasis, frame blocking, windowing, FFT, spectral line energy calculation, and DCT, 44 MFCC features were calculated for each cough sound frame. The detail formula is expressed as follows:

$$MFCC_{i}=\sum_{j=1}^{N}\log(|X_{j}|)\cos(i*(j-0.5)*\frac{\pi }{N})$$ (1)

where i=1,2,…44, *M*_*i*_ denotes the coefficient of MFCC, j=1,2,…, |*X*_*j*_| at equal intervals of frequencies of each frame signal denotes the amplitude of each sampling point.

#### ZCR extraction

We extracted the ZCR features of each cough sound frame by calculating the number of times the sampled value passed through the zero point, as shown in Eqs (2) and (3):

$$Z(n)=\frac{1}{2}(sign(x(n))-sign(x(n-1)))$$ (2)

$$ZCR=\frac{1}{N-1}\sum_{n=1}^{N-1}\left|Z(n)\right|$$ (3)

where x(n) denotes the sample value of the nth sample point, sign(x(n)) denotes the sign function that takes x(n), Z(n) denotes the ZCR value of the nth sample point, and the ZCR of the entire audio signal is calculated.

#### Chroma_cens extraction

Each cough sound frame was Fourier transformed to obtain the frequency-domain signal, which was then divided into 12 equal-width frequency bands. For each bandwidth, the sum of the energies in the bandwidth was calculated and the energy was logarithmic to obtain the chroma index. Finally, a sliding average was performed on each chroma index to obtain the chroma_cens feature. The formula is shown in Eq (4):

$$Chroma\_cens_{t}(p)=\sum_{i\in B(p)}E_{i}(t)H(i-c_{n})w(i-c_{n})$$ (4)

where t denotes the time frame, p denotes the chroma note, B(p) is the frequency band corresponding to chroma note p, *E*_*i*_(*t*) is the amplitude of frequency band i in time frame t, H is a band-pass filter, *c*_*n*_ denotes the reference chroma note, and w is a Hann window function.

#### Conversion method of spectrogram

A Hanning window with a width of 256 [30] was applied to the cough sound fragment. Then, each window signal was fast Fourier-transformed. The mode length of the Fourier coefficient calculation was followed, and the logarithm of the mode length was obtained. Finally, the logarithmic values of all windows were combined according to certain rules to obtain the speech spectrum map. The corresponding formulas are given in Eqs (5) and (6):

$$X_{i}(f)=\sum_{n=0}^{N-1}x_{i}(n)w(n)e^{\frac{-j2\pi nf}{N}}$$ (5)

$$L_{i}(f)=10\log_{10}({\left|X_{i}(f)\right|}^{2})$$ (6)

where f is the frequency, N is the number of points for FFT, w(n) is the window function, j is an imaginary unit, and *X*_*i*_(*f*) is the amplitude spectrum.

### Evaluation indicators

The classification results were compared using the three indicators of accuracy, specificity and sensitivity, and the ROC curve was used to measure the performance of the model. The formulas for the accuracy, specificity, and sensitivity are as follows:

$$Accuracy=\frac{TP+TN}{TP+FN+TN+FP}*100\%$$ (7)

$$Specificity=\frac{TP}{TP+FN}*100\%$$ (8)

$$Sensitivity=\frac{TN}{TN+FP}*100\%$$ (9)

### Cough sound classification model

**Fig 4:**
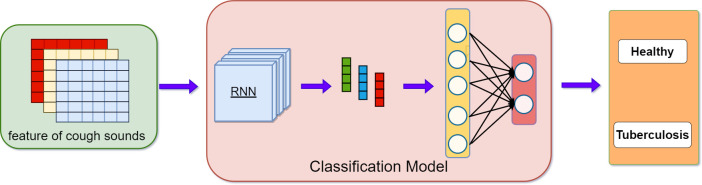
Timing model diagram.

**Fig 5:**
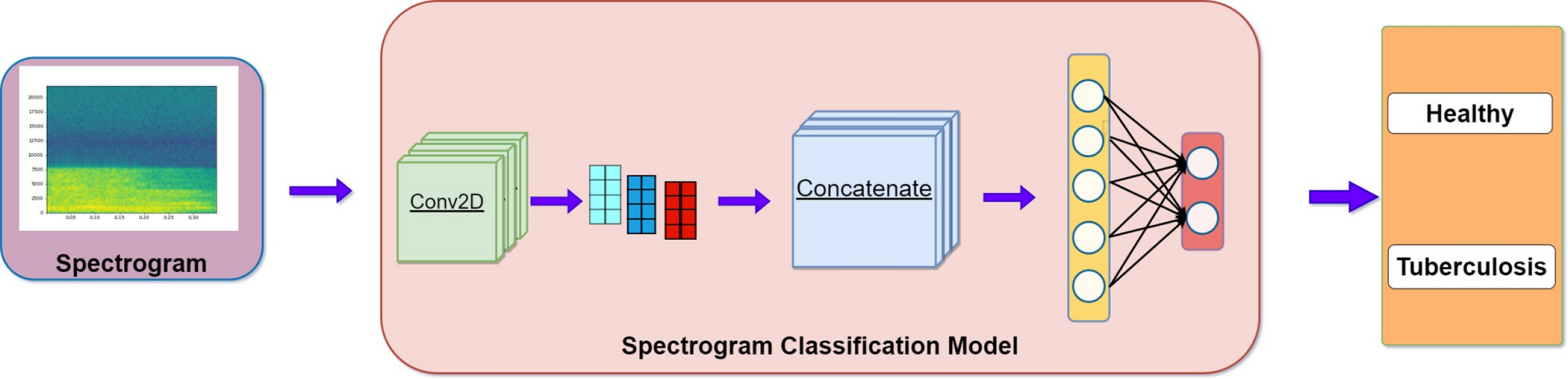
Conv2D model diagram.

LSTM, bidirectional gated recurrent neural network (Bi-GRU) and gated recurrent neural network (GRU), models are commonly used time series models in research of cough sound classification. These were selected for this study. As we previously verified that the best classification results were achieved with five features used simultaneously, the classification performance of the three models was evaluated. The scheme is shown in **Fig 4** and the specific structure is shown in **Table 4**. In addition, two-dimensional convolutional neural network (Conv2D) was a commonly used image classification model. As the data size in the study was restricted, a relatively basic Conv2D model was used. The model diagram is shown in **Fig 5**, and the specific structure is listed in **Table 5**.

**Table 4:** RNN model parameters.

Block Number	Layer Name	Model hyperparameter	Output Shape	Params
Block 1	Bidirectional (LSTM)	units=32, return_sequences=True	(None, 59, 64)	16384
	Bidirectional (LSTM)	units=32, return_sequences=True	(None, 59, 64)	24832
	Flatten		(None, 3776)	0
	Dropout	0.5	(None, 3776)	0
	Dense	128, activation=’relu’	(None, 128)	483456
	BatchNormalization		(None, 128)	512
	Dense	1, activation=’sigmoid’	(None, 1)	129

The naming of the network layer implies that the network layer is a common module of the later model, or the network layer has a special meaning.

**Table 5:** Convolutional neural network parameters.

Block Number	Layer Name	Model hyperparameter	Output Shape	Params
Block 2	Conv2D	filters=16,kernel_size=3,strides=2, padding=’same’activation=’relu’	(None,120, 160, 16)	448
	MaxPooling2D	pool_size=3,strides=2,padding="same"	(None, 60, 80, 16)	0
	Conv2D	filters=16,kernel_size=3,strides=2,padding=’same’,activation=’relu’	(None, 60, 80, 32)	4640
	MaxPooling2D	pool_size=3,strides=2,padding="same"	(None, 30, 40, 32)	0
	Flatten		(None, 38400)	0
	Dropout	0.4	(None, 38400)	0
	Dense	1024, activation=’relu’	(None, 1024)	39322624
	Dense	512, activation=’relu’	(None, 512)	524800
	Dense	128, activation=’relu’	(None, 128)	65664
	Dense	1, activation=’sigmoid’	(None,1)	129

### Tuberculosis detection model based on feature fusion

**Fig 6:**
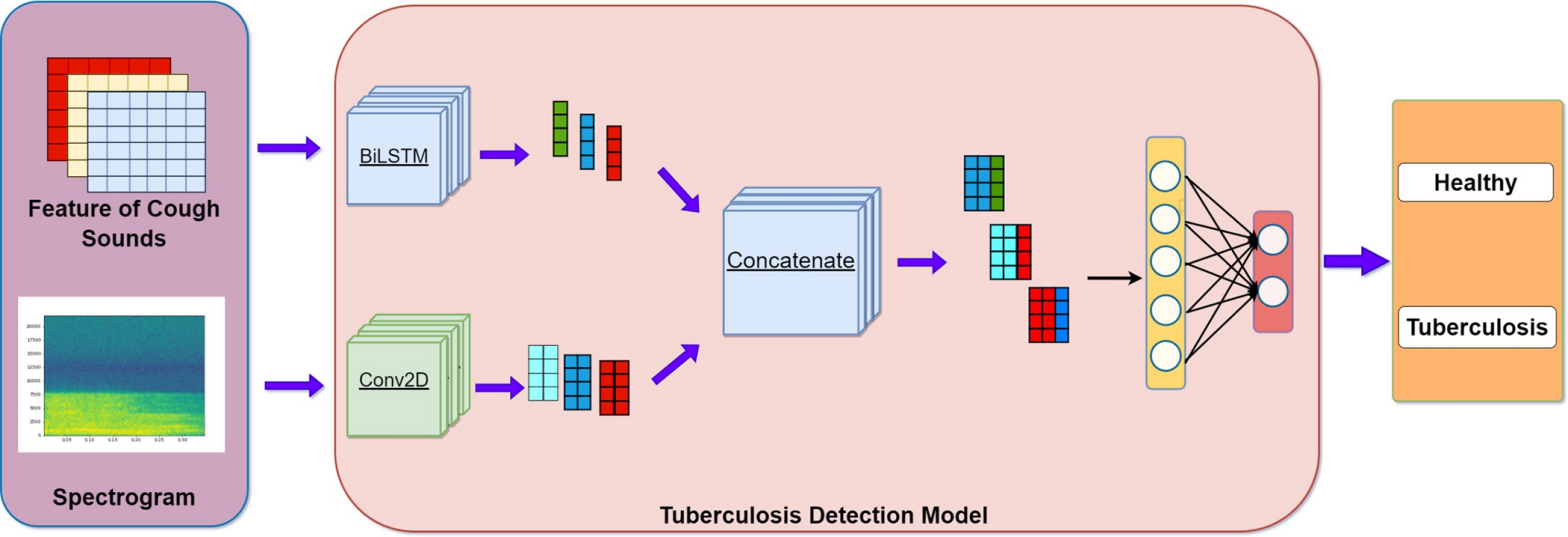
Feature fusion model diagram.

To improve the classification performance, we propose a feature fusion method that fuses the deep features obtained from five features trained by a recurrent neural network with the features extracted from the speech spectral map trained by the convolutional neural network. The performance of the fully connected layer was used for the classification model. The model diagram is shown in **Fig 6** and its specific structure is shown in **Table 6**. Finally, the classification performance was evaluated by comparing specificity, sensitivity, and accuracy, and the model was evaluated using ROC curves.

**Table 6:** Feature fusion model parameters.

Block Number	Layer Name	Model hyperparameter	Output Shape	Params
Block 1				
	Dense	64, activation=’relu’	(None, 64)	241728
Block 2				
Block 3	Concatenate		(None, 192)	0
	Dense	64, activation=’relu’	(None, 64)	12352
	Dense	1, activation=’sigmoid’	(None, 1)	65

## Result

**Fig 7:**
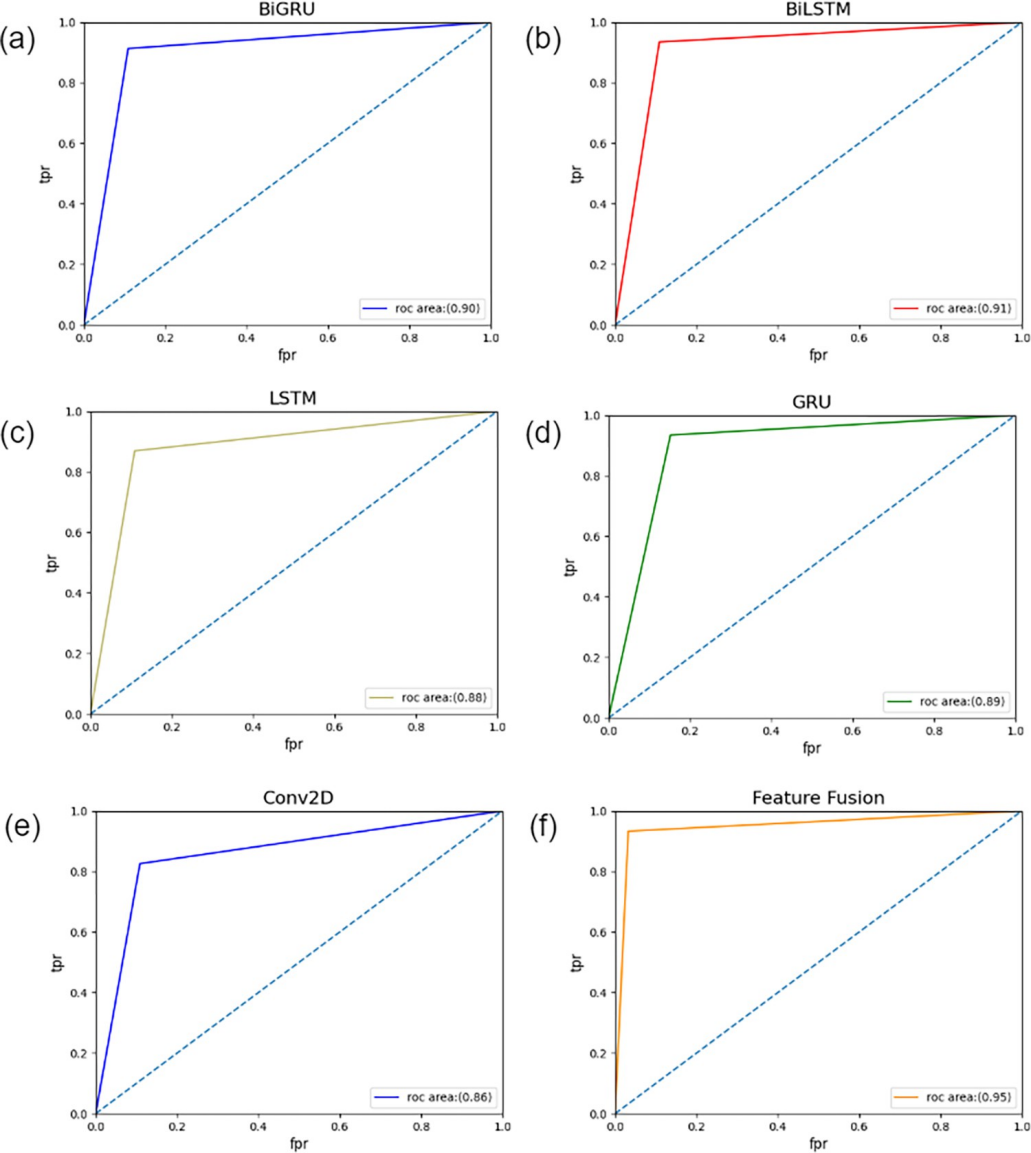
ROC curve of each model, where (a) shows the roc curve of Bi-GRU, (b) shows the roc curve of Bi-LSTM, (c) shows the roc curve of LSTM, (d) the roc curve of Bi-LSTM, (e) shows the roc curve of Covn2D, (f) shows the roc curve of Feature Fusion.

Four models, Bi-LSTM, Bi-GRU, LSTM, and GRU, were trained with the five trained features, MFCC, ZCR, TSE, RMS, and chroma_cens, on each cough sound fragment. The classification results obtained after 10 random partition validation averages are shown in **Table 7**. The results showed that Bi-LSTM performed better than the other three models in terms of accuracy and specificity. However, in terms of sensitivity, the Bi-GRU achieved the best performance. Owing to the relatively small data size, a simpler two-dimensional convolution was used to classify the speech spectrogram. Two models, Bi-LSTM and Conv2D, were selected for training and classification after the feature fusion. The classification results are listed in **Table 8**. The results showed that fusion of the features extracted by both approaches yielded 96.33% accuracy, 94.99% specificity, and 98.96% sensitivity. Compared with the condition with only features used in one way, each parameter showed improvement. The ROC curves of each model are shown in **Fig 7**, where the AUC score of feature fusion was 0.95, and Bi-LSTM reached the best value of 0.92 among all the models. The experimental results show that the inclusion of speech spectrogram features can improve the classification performance.

**Table 7:** Classification results of audio features by 4 models.

Model	Accuracy	Specificity	Sensitivity
Bi-LSTM	**93.99%**	**93.93%**	**92.39%**
Bi-GRU	93.12%	92.88%	93.19%
LSTM	91.31%	92.69%	89.73%
GRU	90.58%	89.52%	91.16%

**Table 8:** Comparison of classification results after adding the Spectrogram.

Model	Accuracy	Specificity	Sensitivity
Bi-LSTM	93.99%	93.93%	92.39%
Conv2D	90.58%	89.52%	91.16%
Bi-LSTM+ Conv2d	**96.33%**	**94.99%**	**98.13%**

## Discussion

A comparative study was conducted to explore artificial intelligence (AI) classification for detecting TB through cough sounds. The present study, with a data size relatively larger compared to those works published in the literature, examined both traditional and deep learning features individually and in the combination. Several AI classification models were evaluated for comparison. The study has limitations, mainly associated with the data size issue. Compared with those in the image domain of deep learning, the data size may be less sufficient for deep learning to achieve high accuracy and robustness. The data of this study was obtained under the control of environmental noise, and its application in practice may be interfered with environmental noise. In addition, there could be variation when applying this method to different populations across various regions. Therefore, further investigation into these potential differences should be conducted. Moreover, how an average of three coughs from the same subject on the research results should be evaluated. Despite these challenges, the results indicate that cough-based tuberculosis screening could become a viable community screening tool in the future. It offers the advantages of high accuracy and low cost for primary screening.

## Conclusion

This study presents a novel screening technique for TB within an existing setting. It employs a feature fusion-based approach to differentiate cough sounds between patients with TB and those without respiratory diseases. When evaluated on parameters such as accuracy, specificity and sensitivity, the proposed method outperforms the currently used PPD screening method. Considering the factors like costs, expenditure, and application scenario, this new method has great potential for use. In conclusion, this technique is suitable for primary community TB screening, offering significant cost benefits and convenience compared with existing TB screening methods.
